# Change in lifestyle behaviors and diabetes risk: evidence from a population-based cohort study with 10 year follow-up

**DOI:** 10.1186/s12966-017-0489-8

**Published:** 2017-03-29

**Authors:** Adina L. Feldman, Gráinne H. Long, Ingegerd Johansson, Lars Weinehall, Eva Fhärm, Patrik Wennberg, Margareta Norberg, Simon J. Griffin, Olov Rolandsson

**Affiliations:** 1 MRC Epidemiology Unit, Institute of Metabolic Science, University of Cambridge, Box 285, Cambridge Biomedical Campus, Cambridge, CB2 0QQ UK; 2 Department of Odontology, Umeå University, 901 87 Umeå, Sweden; 3 Department of Public Health and Clinical Medicine, Epidemiology and Global Health, Umeå University, 901 87 Umeå, Sweden; 4 Department of Public Health and Clinical Medicine, Family Medicine, Umeå University, 901 87 Umeå, Sweden; 5 The Primary Care Unit, Institute of Public Health, University of Cambridge, Cambridge Biomedical Campus, Cambridge, CB2 0SR UK

**Keywords:** Diabetes Mellitus, Health Behaviour, Life Style, Epidemiology, Public Health

## Abstract

**Background:**

Promoting positive changes in lifestyle behavior in the whole population may be a feasible and effective approach to reducing type 2 diabetes burden, but the impact of population shifts of modifiable risk factors remains unclear. Currently most of the evidence on modifiable lifestyle behavior and type 2 diabetes risk on a population level comes from studies of between-individual differences. The objective of the study was to investigate the association and potential impact on disease burden for within-individual change in lifestyle behavior and diabetes risk.

**Methods:**

Population-based prospective cohort study of 35,680 participants aged 30–50 at baseline in 1990–2003 in Västerbotten County, Sweden (follow-up until 2013). Five self-reported modifiable lifestyle behaviors (tobacco use, physical activity, alcohol intake, dietary fiber intake and dietary fat intake) were measured at baseline and 10 year follow-up. Lifestyle behaviors were studied separately, and combined in a score. Incident diabetes was detected by oral glucose tolerance tests. Multivariate logistic regression models and population attributable fractions (PAF) were used to analyze the association between change in lifestyle behavior between baseline and 10 year follow-up, and risk of incident diabetes.

**Results:**

Incident diabetes was detected in 1,184 (3.3%) participants at 10 year follow-up. There was a reduced diabetes risk associated with increase in dietary fiber intake, odds ratio (OR) 0.79 (95% confidence interval (CI) 0.66, 0.96) for increase of at least one unit standard deviation (3.0 g/1,000 kcal) of the baseline distribution, PAF 16.0% (95% CI 4.2, 26.4%). Increase in the lifestyle behavior score was associated with reduced diabetes risk, OR 0.92 (95% CI 0.85, 0.99) per unit increase of the score.

**Conclusions:**

These results support a causal link between lifestyle behavior and type 2 diabetes incidence. A small shift in lifestyle behaviors, in particular intake of dietary fiber, has the potential to reduce diabetes burden in the population and might be a suitable target for public health intervention.

**Electronic supplementary material:**

The online version of this article (doi:10.1186/s12966-017-0489-8) contains supplementary material, which is available to authorized users.

## Background

Randomized trials of intensive lifestyle behavioral interventions targeting individuals with impaired glucose tolerance (IGT) have shown that risk of progression to type 2 diabetes can be reduced by half [1]. The UK National Health Service (NHS) will employ the principle strategies from these trials in high risk populations in the National NHS Diabetes Prevention Programme which was recently launched [2]. While it is important to target this high risk population, the approach is limited by difficulty in systematically identifying individuals with IGT as this requires large-scale screening by oral glucose tolerance tests (OGTT). Furthermore, uptake of intensive lifestyle behavior interventions among people with IGT is generally low [3, 4]. Additionally, from a public health perspective, the population with IGT constitutes a minority of the overall population at risk [5]. Thus, potentially greater benefit may be achieved by shifting overall population distribution of risk factors [6–8]. If successful, this approach may decrease the proportion of the population at relatively high risk as well as reduce the overall burden of type 2 diabetes and other common chronic diseases.

In prospective cohort studies it has been shown that between-individual differences in meeting lifestyle behavior recommendations for diet [9, 10], alcohol consumption [11], physical activity/sedentary behavior [12, 13], smoking [14] or a combination of risk factors [15–17], at one time-point (baseline) is inversely associated with risk of incident diabetes. It has also been shown in US data that within-individual changes in diet quality over time are associated with reduced diabetes risk [18]. This suggests that those who change behavior to meet more lifestyle recommendations could reduce their risk of diabetes, but the extent to which this is the case is unknown. In fact, there is little data to inform policy decisions about interventions in high risk individuals vs. general populations. We aimed to quantify the impact of feasible changes in lifestyle behavior in the adult population on risk of type 2 diabetes.

## Methods

### Study population

The Västerbotten Intervention Programme (VIP) was initially established in 1985 as a community and individual-level programme aimed to reduce the morbidity and mortality from cardiovascular disease in Northern Sweden [19]. The programme has been described in detail previously [19]. Briefly, residents in Västerbotten county are eligible to be invited for standardized health examinations including OGTT to their primary care center during the year of their 30th (until 1995), 40th, 50th and finally 60th birthday. For individuals found to have a BMI ≥30 kg/m^2^ the recommendation is that the attending health care practitioner provides counselling concerning lifestyle changes aimed at risk factor reduction, and those found to have IGT are referred for a follow-up visit with a nurse, generally every second year [19]. At every visit participants are further asked to complete a comprehensive questionnaire that covers among other things lifestyle behavior, health, and psychosocial situation. Baseline participation rate over the study period has ranged between 48 and 67% [20].

For the present study we used the data collected as part of VIP to conduct an observational prospective cohort study. Eligible study individuals were all VIP participants first included between 1990 and 2003 at age 30, 40 or 50, and who participated in at least one 10 year follow-up. In total, 52,889 participants were eligible at baseline. After excluding those who had prevalent diabetes (*n* = 1,280), missing baseline OGTT (*n* = 433) or were lost to follow-up (*n* = 14,980, 29.6%), 36,196 participants with 10 year follow-up remained. As the exposure was change in lifestyle behavior between baseline and 10 year follow-up, cases who self-reported diabetes at follow-up would have experienced the outcome before the second measurement of the exposure. Thus, to limit bias due to potential reverse causality and differential misreporting we excluded participants who self-reported a diabetes diagnosis at 10 year follow-up (*n* = 487) from the main analysis. We further excluded those who at follow-up had missing OGTT (*n* = 29), leaving a total study population of 35,680 participants.

### Assessment of diabetes

The outcome was an incident diabetic OGTT result at 10 year follow-up based on capillary plasma samples, defined as a fasting glucose > =7.0 mmol/L or 2-h glucose of > =12.2 mmol/L [21, 22]. For descriptive purposes we also defined IGT as fasting glucose <7.0 mmol/L and 2-h glucose between > =8.9 and <12.2 mmol/L, as well as Impaired Fasting Glycaemia (IFG) as fasting glucose between > =6.1 and <7.0 mmol/L and 2-h glucose <8.9 mmol/L.

### Assessment of lifestyle behavior

The assessment and definitions of lifestyle behavior has been described in detail previously [17]. The selection of lifestyle behavior recommendations was based on those included in the Finnish Diabetes Prevention Study (DPP) [23] and previous studies of overall lifestyle behavior in association with diabetes [15–17]. Briefly, lifestyle behaviors were measured at baseline and 10 year follow-up in VIP using the same questionnaire [19]. The measurements were then converted into dichotomous achievement of recommendations (yes/no status) according to absolute cut-offs based on targets from DPP [23], observed benefits for diabetes risk from observational studies of lifestyle behaviors [10, 11, 13, 14] or previous studies of lifestyle behavior recommendations and diabetes risk [16, 17].

Tobacco use was assessed by self-reported smoking and use of Swedish moist snuff in the VIP questionnaire (recommendation: no current tobacco use) [14]. Occupational and leisure-time physical activity was assessed using the validated short EPIC-PAQ questionnaire [24] and participants were categorised into four groups ranging from inactive to active (recommendation: moderately active or active) [13]. Dietary fiber, fat and alcohol intake were assessed using a modified version of the validated Northern Sweden Food Group Frequency Questionnaire (FFQ) with 64–84 items [25]. Reported frequencies of consumption were converted to number of intakes per day and multiplied by a portion size to derive daily energy and nutrient intakes calculated in kcal or grams (g)/day, respectively. For alcohol intake the cut-off was >0.0 and ≤20.0 g ethanol/day [11], for dietary fiber intake the cut-off was ≥15.0 g/1,000 kcal of total daily energy intake (equivalent to 4,184 J) [23] and for fat intake the cut-off was <30.0% of total daily energy intake [23]. Due to the observed higher risk of diabetes among non-drinkers (i.e. those consuming 0.0 g ethanol/day) compared to moderate drinkers [11], non-drinkers were considered not to achieve the recommended lifestyle behavior. All categorised lifestyle behaviors were added together to produce a lifestyle behavior score ranging from 0 to 5 achieved recommendations.

### Assessment of other variables

Co-variates were chosen to capture the potential confounding of socio-economic status and family history of diabetes on the association between propensity to change behavior and diabetes risk. Height and weight were measured in light clothing at the health examination and body mass index (BMI) was calculated as weight in kilograms divided by height in meters squared. Marital status was dichotomised as single/divorced/separated/widowed or married/living with partner. Family history of diabetes was defined as presence of diabetes in any parent or sibling. Educational level was categorised as primary (mandatory), any secondary or any tertiary. These variables were self-reported at baseline in the VIP questionnaire.

### Statistical analysis

The association between the outcome, type 2 diabetes at 10 year follow-up, and within-individual change in achievement of lifestyle behavior recommendations was assessed using logistic regression generating odds ratios (OR) and 95% confidence intervals (CI). Two models were used to analyze change in achievement status of each lifestyle behavior recommendation; 1) With adjustment for the co-variates sex, age at baseline (30, 40 or 50), educational level, calendar year at baseline (continuous), marital status, family history of diabetes and BMI at baseline; 2) With additional mutual adjustment for change in achievement of all lifestyle behavior recommendations. Change in lifestyle behavior was defined as either categorical change in achievement status (yes/no) of recommendations between baseline and 10 year follow-up, change in unit standard deviation (SD) of the baseline distribution for continuous measures (dietary fiber, fat, alcohol intake), or as change in unit of the scale compared to baseline for ordinal measures (physical activity). Categorical change in lifestyle behavior was modeled by including the recommendation achievement status at baseline and follow-up with an interaction term between the time-points; each lifestyle recommendation was stratified by its achievement status at baseline, and within each strata change in achievement was compared to no change in achievement. Change in the lifestyle behavior score was modelled categorically with no change in total number of achieved recommendations as the reference level and continuously as change in unit of scale compared to baseline, adjusting for baseline lifestyle behavior score, sex, age at baseline (30, 40 or 50), educational level, calendar year at baseline (continuous), marital status, family history of diabetes and BMI at baseline.

We compared a series of nested models using the likelihood ratio test to assess model fit. The best fit was found to be when including baseline BMI as a continuous variable with a quadratic term.

To assess the impact of changes in lifestyle behavior on diabetes burden in the population we estimated population attributable fractions (PAF) with 95% CI using the “punafcc” command in Stata [26]. The command estimates PAFs using the formula (pd*((*OR*-*1*)/*OR*)) where *pd* is the proportion incident diabetes cases with the exposure [27]. PAFs were based on the maximally adjusted models and calculated for those who improved achievement of lifestyle behavior recommendations between baseline and 10 year follow-up ≥1 unit or ≥1 SD of the baseline distribution compared to the modeled counterfactual which was those who improved less or declined in achievement. To compare baseline characteristics between the study population and participants lost to follow-up we used t-tests for continuous variables and Pearson’s χ^2^ for categorical variables. We conducted two sensitivity analyses; 1) to test whether any single lifestyle behavior drove the association between change in lifestyle behavior and diabetes risk we removed each contributing lifestyle behavior in turn from the lifestyle behavior score and compared results for the analysis of continuous change in lifestyle behavior score and diabetes risk; 2) to assess the impact of excluding self-reported diabetes cases at 10 year follow-up we repeated analyses with these cases retained in the sample. All data were analyzed using Stata v. 13 for Windows.

## Results

Diabetes was newly detected in 1,184 participants at follow-up, constituting 3.3% of the study population (Table 1). When including the participants who self-reported diabetes at follow-up (*n* = 487) and were thus excluded from the study population for main analyses, the cumulative incidence proportion of diabetes in the cohort at 10 year follow-up at age 40–60 years was 4.6%.

**Table 1:** Descriptive statistics of study population including all participants with 10 year follow-up and free of prevalent diabetes, Vӓsterbotten Intervention Programme 1990–2013

		Baseline		10 year follow-up	
Total (n, %)		35,680	100.0	-	
Age at baseline, years (n, %)					
30		5,214	14.6	-	
40		15,012	42.1	-	
50		15,454	43.3	-	
Sex (n, %)					
Men		16,693	46.8	-	
Women		18,987	53.2	-	
Year at baseline (n, %)					
1990–1994		13,301	37.3	-	
1995–1999		16,327	45.8	-	
2000–2003		6,052	17.0	-	
Education (n, %)					
Primary		14,047	39.4	-	
Any secondary		12,268	34.4	-	
Any tertiary		9,122	25.6	-	
Marital status (n, %)					
Single/Widowed/Divorced		5,776	16.2	-	
Married/Partner		29,609	83.0	-	
Family history of diabetes (n, %)		6,032	16.9	-	
BMI, kg/m^2^ (mean, SD)		25.1	3.8	-	
Glucose concentration, mmol/L in capillary plasma (mean, SD)					
Fasting		5.3	0.6	5.5	0.9
2-h value		6.4	1.3	6.8	1.8
Type 2 diabetes based on OGTT (n, %)		-		1,184	3.3
Impaired Glucose Tolerance (n, %)		956	2.7	2,408	6.7
Impaired Fasting Glycaemia (n, %)		2,130	6.0	3,744	10.5
Complete lifestyle behavior data available (n, %)		32,034	89.8	30,553	85.6
Complete lifestyle behavior and co-variate data available (n, %)		-		27,270	76.4
Tobacco use (n, %)					
Current smokers		7,531	21.1	4,519	12.7
Current snuff users		5,222	14.6	5,322	14.9
Recommendation met	Non-users/past users	23,547	66.0	25,087	70.3
Recommendation not met	Current users	11,467	32.1	9,159	25.7
Physical activity (n, %)					
Inactive		9,437	26.4	8,402	23.5
Moderately inactive		8,079	22.6	6,805	19.1
Moderately active		10,204	28.6	10,126	28.4
Active		6,868	19.2	7,887	22.1
Recommendation met	Active/moderately active	17,072	47.8	18,013	50.5
Recommendation not met	Inactive/moderately inactive	17,516	49.1	15,207	42.6
Total energy intake, kcal (mean, SD)		1843.5	597.8	1641.3	544.0
Dietary fiber intake, g/1,000 kcal (mean, SD)		10.5	3.0	11.6	3.4
Recommendation met	≥15.0 g/1,000 kcal (n, %)	2,488	7.0	5,522	15.5
Recommendation not met	<15.0 g/1,000 kcal (n, %)	31,000	86.9	28,619	80.2
Fat intake, %total energy (mean, SD)		34.5	6.2	34.3	6.8
Recommendation met	<30.0% of total energy (n, %)	7,662	21.5	8,933	25.0
Recommendation not met	≥30.0% of total energy (n, %)	25,826	72.4	25,208	70.7
Alcohol intake, g ethanol/day (mean, SD)		3.9	4.3	4.5	4.9
Non-drinkers (n, %)		1,922	5.4	2,221	6.2
Recommendation met	>0.0 and <20.0 g/day (n, %)	32,869	92.1	32,240	90.4
Recommendation not met	0.0 or ≥20.0 g/day (n, %)	2,270	6.4	2,769	7.8
Lifestyle behavior score (median, IQR)		2	2 to 3	3	2 to 3
Change between baseline and 10 year follow-up (mean, SD)		-		0.2	1.0

Detailed missing data: Education, *n* = 243; Marital status, *n* = 295; Family history of diabetes, *n* = 447; BMI, *n* = 132; Tobacco use, *n* = 666 (BL), *n* = 1,434 (10 year follow-up); Physical Activity, *n* = 1,092 (BL), *n* = 2,460 (10 year follow-up); Alcohol intake, *n* = 541 (BL), *n* = 671 (10 year follow-up); Total energy/Fiber/Fat intake, *n* = 2,192 (BL), *n* = 1,539 (10 year follow-up)

*BMI* body mass index, *IQR* inter-quartile range, *OGTT* oral glucose tolerance test, *SD* Standard deviation

Overall, the proportion of participants who achieved individual lifestyle recommendations increased for tobacco use by 4.3%, for physical activity by 2.7%, for dietary fiber intake by 8.5% and for fat intake by 3.5% (Table 1). Alcohol intake showed a trend in the opposite direction with a small decrease of 1.7% in the proportion who achieved the recommendation; the proportion of non-drinkers was 0.8% higher at follow-up compared to baseline (Table 1).

There was a reduced diabetes risk associated with increase in dietary fiber intake; OR 0.86, (95% CI 0.78, 0.94) per increase of unit SD of the baseline distribution (3.0 g/1,000 kcal of total energy) (Table 2). When comparing those who increased ≥1 SD vs. those who did not, the OR was 0.79 (95% CI 0.66, 0.95), which is equivalent to a PAF of 16.0% (95% CI 4.2, 26.4%). For increasing fiber intake to reach the recommendation of ≥15.0 g/1,000 kcal the association with diabetes risk was in the same direction but not significant; OR 0.89 (95% CI 0.70, 1.14) (Table 3). The associations between diabetes risk and change in physical activity and fat intake were in the expected directions but not significant, OR 0.89 (95% CI 0.73, 1.09) and 0.92 (95% CI 0.74, 1.15) for improvement in recommendation achievement, respectively. There was no apparent association between changes in tobacco use or alcohol intake and diabetes risk (Table 3). Mutually adjusting for all lifestyle behaviors (Model 2) did not significantly improve fit vs Model 1 for tobacco use, physical activity, dietary fiber or fat intake (*p* > 0.05) but it did for alcohol intake (*p* = 0.017) (data not shown).

**Table 2:** Association between type 2 diabetes risk and improvement of lifestyle behaviour between baseline and 10-year follow up

		Model 1^a^		Model 2^b^			
Lifestyle behaviour	Change	OR	95% CI	OR	95% CI	PAF (%)	95% CI
Physical activity	Continuous increase	0.95	0.89, 1.01	0.98	0.91, 1.05	-	
	Increase ≥1 point	0.89	0.77, 1.03	0.96	0.82, 1.13	2.7	−8.3, 12.5
Dietary fiber intake	Continuous increase	0.86	0.80, 0.92	0.86	0.78, 0.94	**-**	
	Increase ≥1 SD^c^	0.80	0.68, 0.93	0.80	0.66, 0.95	16.0	4.1, 26.4
Fat intake	Continuous decrease	0.94	0.88, 1.00	1.02	0.94, 1.11	-	
	Decrease ≥1 SD^d^	0.91	0.76, 1.09	0.94	0.76, 1.16	5.0	−13.2, 20.3
Alcohol intake	Continuous decrease	1.00	0.93, 1.07	1.01	0.93, 1.10	-	
	Decrease ≥1 SD^d^	0.89	0.65, 1.21	0.93	0.66, 1.32	6.2	−30.4, 32.6
Lifestyle behaviour score^e^	Continuous increase	-		0.92	0.85, 0.99	-	
	Increase ≥1 unit	-		0.92	0.78, 1.07	5.5	−4.2, 14.3

PAFs calculated based on Model 2. Continuous measures are estimates per unit standard deviation of the baseline distribution, except physical activity which is an ordinal 4-point scale

^a^ Model adjusted for baseline absolute level of behaviour, baseline BMI, sex, marital status, education at baseline in 3 categories, calendar year at baseline, family history of diabetes yes/no, age group at baseline (30, 40 or 50)

^b^ Model additionally mutually adjusted for achievement status of all recommendations at baseline and 10 year follow-up

^c^ Reference group is all who did not change, increased less than one SD or decreased their intake

^d^ Reference group is all who did not change, decreased less than one SD or increased their intake

^e^ Change in the lifestyle behaviour score ranges from − 5 to +5. Model adjusted for baseline number of total achieved lifestyle behaviour recommendations, baseline BMI, sex, marital status, education at baseline in 3 categories, calendar year at baseline, family history of diabetes yes/no, age group at baseline (30, 40 or 50)

*BMI* body mass index, *CI* confidence interval, *OR* odds ratio, *PAF* population attributable fraction, *SD* standard deviation

**Table 3:** Association between type 2 diabetes risk and maintenance or improvement of lifestyle recommendation achievement between baseline and 10-year follow up

	Recommendation met			Total	Diabetes cases at 10 year follow-up		Model 1^a^		Model 2^b^	
Lifestyle behaviour	Baseline	10 year follow-up	Change	n	n	%	OR	95% CI	OR	95% CI
Tobacco use	Yes	Yes	Maintenance	21,695	675	3.1	1.00	ref	1.00	ref
*Recommendation*:	Yes	No	No maintenance	1,362	41	3.0	0.92	0.66, 1.29	0.94	0.64, 1.38
*No current smoking*/*snuff*	No	No	No improvement	7,597	290	3.8	1.00	Ref	1.00	ref
	No	Yes	Improvement	3,069	128	4.2	1.09	0.87, 1.36	1.04	0.81, 1.35
Physical Activity	Yes	Yes	Maintenance	11,624	319	2.7	1.00	ref	1.00	ref
*Recommendation*:	Yes	No	No maintenance	4,317	129	3.0	1.04	0.84, 1.29	1.00	0.79, 1.26
*Moderately active*/*Active*	No	No	No improvement	10,508	393	3.7	1.00	Ref	1.00	ref
	No	Yes	Improvement	5,900	184	3.1	0.88	0.73, 1.06	0.89	0.73, 1.09
Dietary fiber intake	Yes	Yes	Maintenance	1,259	37	2.9	1.00	ref	1.00	ref
*Recommendation*:	Yes	No	No maintenance	1,133	45	4.0	1.34	0.84, 2.12	1.38	0.80, 2.37
>*15.0 g*/*1*,*000 kcal of total energy*	No	No	No improvement	25,824	836	3.2	1.00	ref	1.00	ref
	No	Yes	Improvement	4,001	121	3.0	0.83	0.68, 1.02	0.89	0.70, 1.14
Fat intake	Yes	Yes	Maintenance	3,415	102	3.0	1.00	ref	1.00	ref
*Recommendation*:	Yes	No	No maintenance	3,948	150	3.8	1.25	0.96, 1.63	1.26	0.93, 1.71
<*30.0* % *of total energy*	No	No	No improvement	19,838	631	3.2	1.00	ref	1.00	ref
	No	Yes	Improvement	5,016	156	3.1	0.91	0.75, 1.09	0.92	0.74, 1.15
Alcohol intake	Yes	Yes	Maintenance	31,125	1,008	3.2	1.00	ref	1.00	ref
*Recommendation*:	Yes	No	No maintenance	1,229	49	4.0	1.06	0.78, 1.43	1.13	0.79, 1.59
>*0.0* & <*20.0 g*/*day*	No	No	No improvement	1,488	52	3.5	1.00	ref	1.00	ref
	No	Yes	Improvement	728	25	3.4	1.11	0.67, 1.84	1.17	0.65, 2.10

^a^ Model adjusted for baseline BMI, sex, marital status, education at baseline in 3 categories, calendar year at baseline, family history of diabetes yes/no, age group at baseline (30, 40 or 50)

^b^ Model additionally mutually adjusted for achievement status of all recommendations at baseline and 10 year follow-up

*BMI* body mass index, *CI* confidence interval, *OR* Odds ratio

**Fig. 1:**
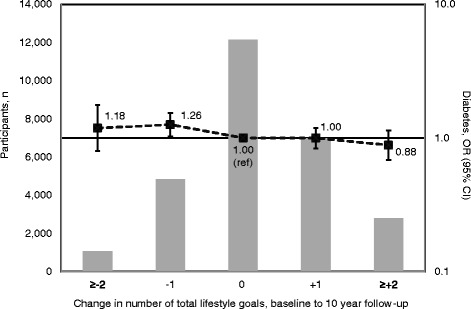
Distribution of change in total number of achieved lifestyle recommendations between baseline and 10 year follow-up (bars, left axis) and association between change and diabetes risk at 10 year follow-up (forest plot, right axis). ORs adjusted for baseline number of total achieved lifestyle behavior recommendations, BMI, sex, marital status, education in 3 categories, calendar year, family history of diabetes yes/no, age group (30, 40 or 50). *CI* confidence interval, *OR* odds ratio

Overall, *n* = 14,327 (40.2%) participants improved in achieving at least one lifestyle behavior recommendation during follow-up and *n* = 10,249 (28.7%) failed to maintain the achievement of at least one recommendation. On average the participants achieved 0.2 more recommendations at 10 year follow-up compared to baseline, and the median number of recommendations achieved increased from 2 (IQR 2, 3) at baseline to 3 (IQR 2, 3) at 10 year follow-up (Table 1). The distribution of change in total number of achieved recommendations is shown in Fig. 1. There was a linear association between change in total number of recommendations achieved and diabetes risk; OR 0.92 (95% CI 0.85, 0.99) per increase of one unit of the lifestyle behavior score (range from − 5 to +5) (Table 2). For increase of one or more total lifestyle behavior recommendations vs. no change or decrease the OR was 0.92 (95% CI 0.78, 1.07) which is equivalent to a PAF of 5.5% (95% CI −4.2, 14.3%). When we removed each behavior in turn from the score and repeated the analysis for linear change in total number of recommendations achieved and diabetes risk; the ORs ranged from 0.90 (95% CI 0.83, 0.98) when removing tobacco, and 0.94 (95% CI 0.85, 1.03) when removing dietary fat intake (data not shown). To assess impact of excluding self-reported diabetes cases at 10 year follow-up we repeated analyses including these participants. Results were unchanged or attenuated for physical activity, fat intake, alcohol intake and lifestyle behavior score. For dietary fiber intake the results were reversed; there was an increased risk of diabetes associated with increased dietary fiber intake; OR 1.13 (95% CI 1.04, 1.22) per increase of one unit SD of the baseline distribution (Additional file 1: Table S1). Cases who self-reported a diabetes diagnosis at 10 year follow-up had on average the same intake of dietary fiber at baseline as newly diagnosed cases (10.5 g/1,000 kcal/day), but there was a substantial difference in change in intake during follow-up as self-reported cases increased 3 times as much as newly diagnosed cases; 2.84 vs. 0.90 g/1,000 kcal/day (data not shown). The change in dietary fiber intake among newly diagnosed cases was comparable to the change in the whole study population; 1.1 g/1,000 kcal/day (Table 1).

Overall, 29.6% of diabetes-free participants at baseline were unable to be followed-up (includes deaths and migrations from the study area). Participants were less likely to be followed-up at 10 years in the younger compared to older age groups; 34.7, 29.6 and 27.6% of those aged 30, 40 and 50 at baseline, respectively, were lost to follow-up. More men than women (31.3 vs. 28.0%), and more participants with tertiary compared to only primary education (32.6 vs. 27.1%) were lost to follow-up. Participants were also more likely to be lost to follow-up if they had IGT (40.0%) or IFG (35.6%) at baseline. We tested the difference in average change in lifestyle behavior score between baseline and 10 year follow-up for participants with IGT or IFG compared to those with normal glucose at baseline and found no significant differences (*p* = 0.20 and 0.94, respectively). Participants lost to follow-up had higher average baseline BMI (25.6 vs. 25.1 kg/m^2^), were more likely to use tobacco (37.7 vs. 32.1%) but overall achieved the same total number of lifestyle behavior recommendations at baseline, median 2 (IQR 2, 3).

## Discussion

In this population-based cohort of more than 35,000 participants we have shown that changing behavior to achieving more healthy lifestyle recommendations, or maintaining a healthy lifestyle, can reduce diabetes risk. The largest impact was observed for dietary fiber intake, but there was also a protective effect of maintaining or improving overall healthy lifestyle behavior in terms of diet, physical activity and tobacco use.

Few data are available to study shifts in population distributions of risk factors for diabetes. Using these data we can compare the potential impact of a high risk intervention approach [1, 23, 28] to a population shift in lifestyle behaviors on the burden of diabetes. Among the incident diabetes cases at 10 year follow-up 12.9% had had IGT at baseline (*pd*). Assuming all participants would undertake intensive lifestyle interventions the estimated reduction in diabetes in this group would be by half (hazard ratio 0.51 [1]). Using these figures and the formula (pd*((*RR*-*1*)/*RR*)) [27] we estimated the PAF of diabetes for treating all individuals with IGT to 6.3%. As we have shown, this is less than half of the proportion of diabetes cases that can be prevented if everyone increased their dietary fiber intake by 3.0 g/1,000 kcal of total energy per day. For an adult, this amount is equivalent to about 5–7 g of dietary fiber which is roughly the amount found in 2 pears, or a serving of bran flakes, or 100 g of hazelnuts [29]. It was beyond the scope of the present study to compare the impact of different types and sources of dietary fiber but previous studies of between-individual differences in dietary fiber intake and prospective diabetes risk suggest that the strongest association is found for cereal fiber [10, 30]. Dietary fiber intake lowers post-prandial blood glucose [31] and a beneficial effect of dietary fiber on insulin resistance has also been shown in study participants at high risk of diabetes [32, 33]. Compared to the low feasibility of regularly screening whole populations to identify IGT (combined with the knowledge that willingness of individuals with IGT to participate in intensive lifestyle interventions is low [4]), encouraging small increases in intake of readily accessible foods with high fiber may be more feasible, more effective and have a greater impact on population diabetes burden. Reducing diabetes may in turn reduce the burden of cardiovascular morbidity and mortality [34, 35].

Results from previous observational population-based studies on lifestyle behavior and diabetes risk in general populations have largely relied on between-individual differences of exposure measurements from one baseline time-point [9–15, 17]. The causal interpretation of such studies depends on the assumption that the difference in risk attributed to differences in exposure between individuals reflects the impact of change in exposure within individuals. With this study and another recent study on changes in diet quality and diabetes risk [18] this assumption is being tested and the results indicate that it is supported. We may never be able to conclusively prove the causality of an association without conducting a methodologically rigorous randomized trial which is unrealistic for lifestyle behavior in a large general population. However, we may approach better understanding of the mechanism of an association by observationally studying change in exposure within individuals in addition to differences between individuals. Although the present study did not find any association between diabetes risk and change in tobacco use and alcohol intake, and only indications of an association with change in physical activity and fat intake, that does not disprove that these exposures may play an etiological role and be causally associated with diabetes. In the case of smoking, although never smoking is likely associated with lower risk of diabetes compared to ever smoking [14], smoking cessation may increase risk of diabetes in the short term possibly mediated by weight gain and systemic inflammation [36]. The measure of physical activity did not capture changes in light leisure-time activity such as walking or cycling, which may explain the weak association shown. However, as results for change in the lifestyle behavior score showed, although change in individual behaviors may not greatly affect diabetes risk, there may still be an interactive effect, and maintenance or improvement of overall healthy behavior appeared to be protective. The sensitivity analysis showed that no one lifestyle behavior drove the association between change in the score and diabetes risk since removing each lifestyle behavior from the score did not have any great impact on the measure of the association, which indicated that overall healthy lifestyle behavior can be defined in many different ways.

The study has several strengths; most importantly all participants underwent systematic screening for diabetes by OGTT at baseline and 10-year follow-up providing valid ascertainment of both cases and non-cases. Patients in general practice with e.g. high BMI are more likely to be tested for the presence of diabetes than patients with a seemingly low risk profile. Thus, when clinical diagnoses from general practice are used to ascertain diabetes as an outcome in epidemiological studies there is a risk of overestimation of the impact of the risk factor on the outcome (surveillance bias). Further strengths of the present study include measures of actual feasible change in behavior, repeated standardized measures of exposures, the large study population and population-based setting. With regards to the baseline measurements, the long follow-up minimizes bias due to reverse causation.

A limitation of the present study is that diet, alcohol, smoking and physical activity were all self-reported. Self-reported exposures like diet (from FFQ) and physical activity are known to suffer from low validity [24, 25]. This will have caused some misclassification of recommendation achievement status but likely the impact was less than if we had focused on baseline differences between individuals as opposed to change within individuals. Any such bias due to misclassification of exposure is in addition likely to be towards the null. As all participants in VIP underwent health examinations and were enrolled in a community-based programme a greater proportion may have changed to or maintained healthy lifestyle than would have been expected in the absence of such a programme. However, even if participation in VIP affected the relative numbers that changed lifestyle, it should not have affected the effect of changing lifestyle on diabetes risk and consequently the associations observed in the data. As the sensitivity analysis showed, self-reported diabetes cases at follow-up had larger increases in dietary fiber intake than those whose diabetes was newly detected at 10 year follow-up, likely due to changes in lifestyle behavior motivated by a clinical diabetes diagnosis combined with possible over reporting to be in line with dietary advice given to diabetes patients. This resulted in an observed association between increase in dietary fiber intake and increased diabetes risk, the inverse of what was seen when the self-reported cases were excluded. Although participants who self-reported diabetes at follow-up were excluded from the main analyses we cannot exclude residual reporting bias at follow-up, but this potential bias should be limited. More than one quarter of the baseline participants were lost to follow-up and participants with IGT or IFG at baseline were more likely to be lost than those with normal glucose levels. Given the increased risk of diabetes in this group, we may suspect that diabetes was underestimated in the final study population. However, the prevalence of diabetes including self-reported cases at 10 year follow-up was comparable to the estimated prevalence of diabetes in the Swedish population [37]. As there was no difference between change in lifestyle behavior score and IGT/IFG status at baseline, the estimates for risk of diabetes and PAFs may be attenuated but the resulting bias is likely limited.

## Conclusions

In conclusion, these results show that shifting the distribution of lifestyle behaviors, in particular dietary fiber intake, in the adult population could have an important impact on the incidence of diabetes and, as a consequence, cardiovascular morbidity and mortality on a population level [34, 35]. Repeatedly measured objective longitudinal data from large population-based cohorts would help clarify the impact of population shifts of modifiable risk factors on diabetes burden.

## Additional file

Additional file 1: Table S1. Association between diabetes risk and continuous improvement of lifestyle behaviour between baseline and 10‐year follow up. Sensitivity analysis with diabetes cases who self‐reported a diagnosis at 10 year follow‐up (*n* = 487) included as outcome events in the study population. (PDF 93 kb)

## Abbreviations

BMI: Body mass index
CI: Confidence interval
EPIC-PAQ: European prospective investigation of cancer - physical activity questionnaire
FFQ: Food frequency questionnaire
g: Gram
IFG: Impaired fasting glycaemia
IGT: Impaired glucose tolerance
IQR: Inter-quartile range
kcal: Kilocalories
NHS: National Health Service
OGTT: Oral glucose tolerance test
OR: Odds ratio
PAF: Population attributable fraction
SD: Standard deviation
VIP: Västerbotten intervention programme
