# Supplementary material for: Change in lifestyle behaviors and diabetes risk: evidence from a population-based cohort study with 10 year follow-up
Source: Int J Behav Nutr Phys Act. 2017 Mar 29;14:39. doi: 10.1186/s12966-017-0489-8 (PMC5371247; doi:10.1186/s12966-017-0489-8)
Supplement: Additional file 1: Table S1. — Association between diabetes risk and continuous improvement of lifestyle behaviour between baseline and 10‐year follow up. Sensitivity analysis with diabetes cases who self‐reported a diagnosis at 10 year follow‐up (n = 487) included as outcome events in the study population. (PDF 93 kb) [file 12966_2017_489_MOESM1_ESM.pdf]

Supplementary Table 1. Association between diabetes risk and continuous improvement of lifestyle behaviour between baseline and 10-year follow up. Sensitivity analysis with diabetes cases who self-reported a diagnosis at 10 year follow-up (n=487) included as outcome events in the study population.

		Model 2[a]			
Lifestyle behaviour	Change	OR	95% CI	PAF (%)	95% CI
Physical activity	Continuous increase	0.97	0.92, 1.03	-	
	Increase ≥1 point	0.99	0.86, 1.13	0.8	-8.5, 9.4
Dietary fibre intake	Continuous increase	1.13	1.04, 1.22	-	
	Increase ≥1 SD[b]	1.17	1.01, 1.35	-11.9	-24.4, -0.6
Fat intake	Continuous decrease	0.90	0.84, 0.97	-	
	Decrease ≥1 SD[c]	0.91	0.76, 1.09	7.5	-6.9, 20.0
Alcohol intake	Continuous decrease	1.02	0.94, 1.10	-	
	Decrease ≥1 SD[c]	1.10	0.82, 1.47	-9.3	-44.3, 17.2
Lifestyle behaviour score[d]	Continuous increase	0.99	0.93, 1.06	-	
	Increase ≥1 unit	1.02	0.89, 1.17	-1.3	-10.1, 6.9

Continuous measures are estimates per unit standard deviation of the baseline distribution, except physical activity which is an ordinal 4-point scale.

[a] Model adjusted for baseline absolute level of behaviour, baseline BMI, sex, marital status, education at baseline in 3 categories, calendar year at baseline, family history of diabetes yes/no, age group at baseline (30, 40 or 50) and additionally mutually adjusted for achievement status of all recommendations at baseline and 10 year follow-up.

[b] Reference group is all who did not change, increased less than one SD or decreased their intake.

[c] Reference group is all who did not change, decreased less than one SD or increased their intake.

[d] Change in the lifestyle behaviour score ranges from -5 to +5. Model adjusted for baseline number of total achieved lifestyle behaviour goals, baseline BMI, sex, marital status, education at baseline in 3 categories, calendar year at baseline, family history of diabetes yes/no, age group at baseline (30, 40 or 50).

BMI: Body Mass Index, CI: Confidence Interval, OR: Odds ratio, PAF: Population attributable fraction, SD: Standard deviation.
